# Perioperative interdisciplinary optimisation of patients with heart failure undergoing non-cardiac surgery with intermediate or high surgical risk: the rationale and study protocol for the multicentre, randomised interventional PeriOP-CARE HF trial

**DOI:** 10.1007/s00392-025-02626-3

**Published:** 2025-04-29

**Authors:** The PeriOP-CARE HF study group, Götz Friedrich Schmidt, Marit Habicher, Ester Judith Herrmann, Manuel Kenz, Martin Reichert, Christian Koch, Melanie Markmann, Emmanuel Schneck, Birgit Aßmus, Michael Sander, Ulrich Frey, Gülmisal Güder, Patrick Meybohm, Daniel Reuter, Bernd Saugel, Sascha Treskatsch, Peter Ulrich Heuschmann, Carolin Nuernberger, Jens Peter Reese, Jonas Widmann, Viktoria Rücker, Golschan Asgarpur, Saeed Aldarwish, Katharina Beyer, Nikolaus Buchmann, Sven Flemming, Christoph-Thomas Germer, Andreas Greiner, Carla Davina Grundmann, Thilo Hackert, Andreas Hecker, Karim Kouz, Ulf Landmesser, Benjamin L. Löser, Jonas Lehmacher, Sven Märdian, Johannes Tobias Neumann, Alper Öner, Thorsten Riese, Benjamin Sasko, Stefan Störk, Christian Ukena, Thilo Welsch, Franziska Willis, Amelie Zitzmann

**Affiliations:** Giessen, Germany

**Keywords:** Preoperative, Brain natriuretic peptide, Infection, Rehospitalisation, Acute kidney injury, Acute decompensated heart failure

## Abstract

**Aim:**

Chronic heart failure (HF) is a frequent comorbidity in elderly patients undergoing major non-cardiac surgery with increasing prevalence. This trial aims to evaluate a new interdisciplinary, multimodal and individually optimised treatment strategy in patients with established or at risk for HF throughout the entire perioperative period.

**Methods:**

The PeriOP-CARE HF trial is a prospective, multicentre, randomised, controlled and interventional trial. The primary hypothesis is that an interdisciplinary, intersectoral and standardised approach to the preoperative evaluation, optimisation and perioperative management of patients aged ≥ 65 years undergoing non-cardiac surgery with intermediate or high surgical risk and preoperative N-terminal pro-brain natriuretic peptide levels ≥ 450 pg/mL, will reduce postoperative morbidity. The preoperative evaluation includes clinical evaluations by anaesthesiologists and cardiologists, electrocardiography and echocardiography, as well as a discussion of these findings by a perioperative management team, where all involved specialities, including the speciality surgeon, will decide the perioperative treatment strategy for each patient. Intraoperative strategies include individualised haemodynamic optimisation. The interdisciplinary team and specialised HF nurses will screen patients for HF-related postoperative complications. The primary end point will be a composite morbidity end point, comprising any rehospitalisation, acute kidney injury, suspected or proven bacterial infection requiring treatment and acute decompensated HF at postoperative day 90.

**Conclusion:**

The new treatment form can potentially reduce the morbidity burden after major non-cardiac surgery in patients with known or unknown HF. If the PeriOP-CARE HF trial yields positive results, the treatment of patients with HF undergoing major non-cardiac surgery could be considerably improved.

**Trial Registration:**

clinicaltrials.gov: NCT06381427, registered April 24, 2024.

**Supplementary Information:**

The online version contains supplementary material available at 10.1007/s00392-025-02626-3.

## Introduction

Chronic heart failure (HF) is a frequent comorbidity in elderly patients undergoing major non-cardiac surgery [1]. Its overall prevalence is increasing in ageing populations, and its prevalence in patients aged > 65 years is currently estimated at > 10% [2]. Chronic HF is associated with increased postoperative morbidity and mortality. A recent observational study revealed that the incidence of postoperative acute HF is approximately 2.5%, leading to markedly elevated mortality rates of up to 44% [1]. Furthermore, half of the patients with postoperative acute HF are not diagnosed with HF preoperatively. These findings not only indicate the need for careful management of patients with HF requiring non-cardiac surgery but also emphasise the need to identify patients with undiagnosed HF who might be at even greater risk for perioperative complications.

Current European Society of Cardiology (ESC) guidelines on the cardiovascular assessment and management of patients undergoing non-cardiac surgery recommend that patients undergoing major non-cardiac surgery should be carefully examined for signs of HF. However, diagnosing HF based solely on patient’s history and physical examination may be challenging during the preoperative evaluation [3]. Measuring preoperative cardiac biomarkers, such as natriuretic peptides, might help identify patients with unknown HF, assess their perioperative risk and predict their risks of mortality, stroke and cardiovascular adverse events [4]. Therefore, measuring the N-terminal pro-brain natriuretic peptide (NT-pro-BNP) level has a Class IIa recommendation for patients with known cardiovascular disease, cardiovascular risk factors or symptoms suggestive of cardiovascular disease undergoing non-cardiac surgery with intermediate or high surgical risk. Although routine NT-pro-BNP assessment is currently not recommended (Class III recommendation), natriuretic peptides have generally been shown to effectively predict major cardiac adverse events after major non-cardiac surgery in different patient populations [5–8].

Nevertheless, clinically applicable cut-offs triggering distinct actions are lacking, and current guidelines do not recommend any specific perioperative treatment strategies in patients that could be directly derived from a certain preoperative NT-pro-BNP measurement [3, 9]. Therefore, the current European Society of Anaesthesiology and Intensive Care-focused guideline for using cardiac biomarkers in perioperative risk evaluation limits preoperative NT-proBNP-derived management to clinical research because insufficient clinical data are available [9]. Since most postoperative HF-related complications occur during the first days after surgery, optimisation of patients should already begin preoperatively and should be followed by adequate intra- and post-operative management [10].

Therefore, the PeriOP-CARE HF trial aims to combine systematic preoperative screening using a new NT-proBNP cut-off with a standardised preoperative evaluation to provide interdisciplinary, multimodal and individually optimised treatment throughout the entire perioperative period.

## Study design

The PeriOP-CARE HF trial is a prospective, multicentre, randomised, controlled, interventional trial that will be conducted at six university hospitals in Germany: Berlin (Charité, Campus Benjamin Franklin), Giessen, Hamburg, Herne, Würzburg and Rostock. All trial activities will be conducted according to the relevant guidelines and regulations, such as the Declaration of Helsinki and the Good Clinical Practice guidelines. Informed consent to participate will be obtained from all subjects before any trial-specific activities.

**Fig. 1:**
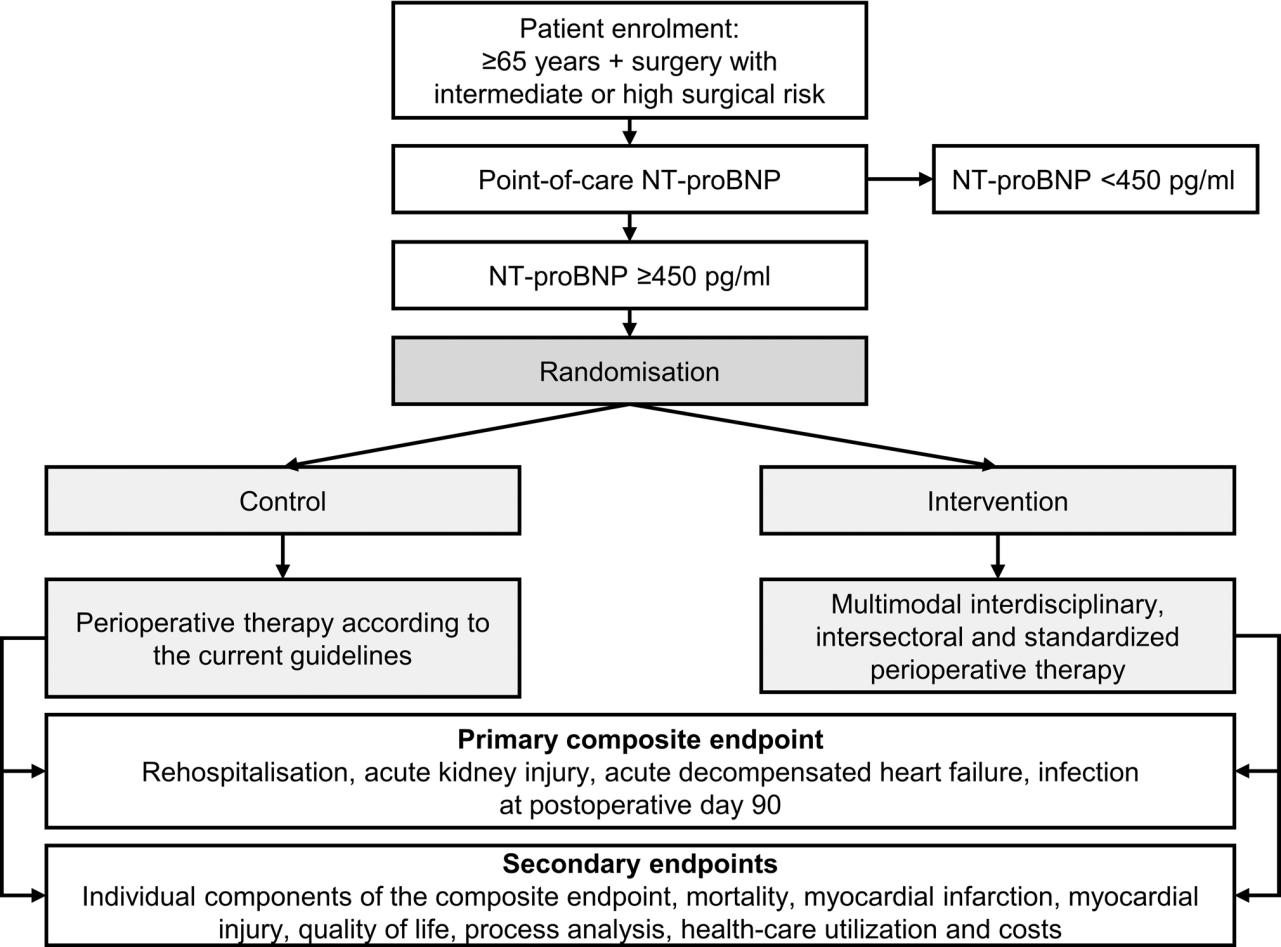
Study flowchart

The primary hypothesis is that an interdisciplinary, intersectoral and standardised approach to the preoperative evaluation, optimisation and perioperative management of patients aged ≥ 65 years undergoing non-cardiac surgery with intermediate or high surgical risk and preoperative NT-proBNP levels of ≥ 450 pg/mL, regardless of preoperative known or unknown HF status, will reduce postoperative morbidity (Fig. 1).

This trial has been approved by the Local Ethics Committee of the Faculty of Medicine at Justus Liebig University Giessen, Germany (approval number: AZ 73/22), as the primary ethical review board for this trial, and all participating centres will obtain approval from their local responsible ethics committees according to individual legislation requirements. This trial has received a full public grant from the Innovationsfonds of the Gemeinsamer Bundesausschuss (grant no. 01NVF22109). The enrolment of the first patient is planned for the end of 2024, and the last patient is expected to complete the follow-up by the mid of 2026. This trial is registered at ClinicalTrials.gov in April 2024 (NCT06381427).

### Study population

The study population will comprise patients aged > 65 years with American Society of Anesthesiologists classification II or higher who will undergo elective major non-cardiac surgery under general anaesthesia. Surgical risk will be assessed according to the current 2022 ESC guidelines on the cardiovascular assessment and management of patients undergoing non-cardiac surgery, and only patients with intermediate or high surgical risk will be included [3]. The exclusion criteria include surgery with an approximated duration of less than 30 min, transplant surgery and other criteria that might interfere with the chosen end points. The inclusion and exclusion criteria are summarised in Table 1.

**Table 1:** Inclusion and exclusion criteria

Inclusion criteria	Exclusion criteria
Age ≥ 65 years	Incision suture time < 30 min
Elective non-cardiac surgery with intermediate or high surgical risk under general anaesthesia	Surgery with general anaesthesia within the past 30 days
ASA ≥ II	Local or regional anaesthesia without general anaesthesia Surgery involving the kidneys (e.g. nephrectomy, partial kidney resection) Chronic kidney disease with eGFR < 15 ml/min or chronical dialysis Surgery utilising cardiopulmonary bypass Emergency surgery Transplant surgery Participation at another interventional trial Insufficient language skills Lacking capacity to consent of patients under legal supervision

*ASA* American Society of Anesthesiologists, *eGFR* estimated glomerular filtration rate

Patients will be screened when they present for routine preoperative evaluation before the scheduled surgery. After written informed consent to participate in this trial is obtained, the patient’s NT-proBNP will be measured in venous blood using a validated point-of-care immunoassay with a measurement range of 60–9,000 pg/mL (proBNP + , cobas h 232; Roche Holding AG, Basel, Switzerland), which yields a result in approximately 10–12 min. If the NT-proBNP level is < 450 pg/mL, further participation in this trial is precluded, and the patients will undergo routine treatment. If the NT-proBNP level is ≥ 450 pg/mL, the patients will undergo 1:1 block randomisation using a computer-generated list implemented in the electronic case report form into a standard care and an intervention care group.

Because evidence for specific NT-proBNP cut-offs triggering distinct treatment strategies is lacking, we conducted a pilot observational study at the anaesthesiology outpatient clinic of the University Hospital of Giessen, where a clinically applicable NT-proBNP cut-off of 450 pg/mL showed a favourable selection of patients at high risk for postoperative morbidity [11]. Specifically, we evaluated the postoperative morbidity of patients with or without known HF, stratified by preoperative NT-proBNP level, which was obtained in the same way and at the same timepoint as it will be in the PeriOP-CARE HF trial.

Patients randomised into the control group will receive routine care according to current guidelines, local standard operating procedures and in line with the attending physicians with no further trial-specific intervention. Patients randomised into the intervention group will receive interdisciplinary, intersectoral, standardised preoperative optimisation and perioperative treatment.

### Intervention

**Fig. 2:**
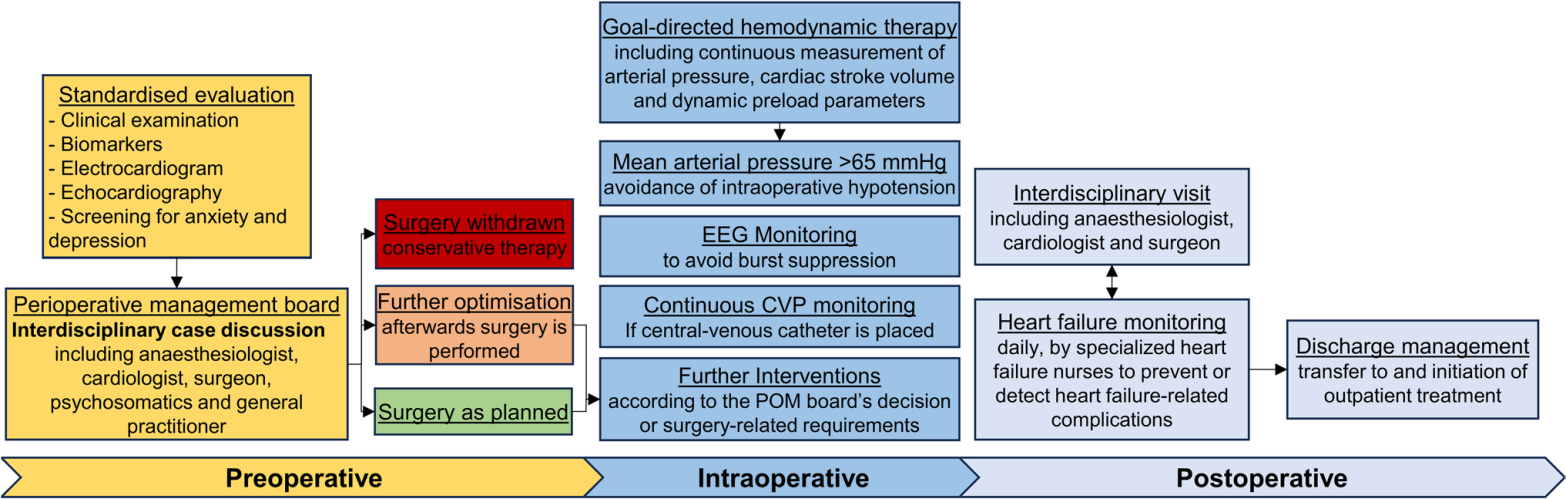
Multimodal, interdisciplinary, intersectoral perioperative interventions in the intervention group. *EEG* electroencephalogram, *CVP* central venous pressure, *POM*: perioperative management

The intervention period covers the entire perioperative period, starting immediately after randomisation into the intervention group and ending at the patients’ hospital discharge after surgery. An overview of the treatment interventions during the different perioperative periods is provided in Fig. 2. In addition to standard care, all patients will receive a standardised preoperative evaluation immediately in the anaesthesia outpatient clinic. Therefore, clinical examination by anaesthesiologists and cardiologists is initiated, followed by electrocardiography (ECG) and transthoracic echocardiography. Patients will be screened for depression and anxiety using the Personal Health Questionnaire-9 (PHQ-9) and Generalised Anxiety Disorder-7 (GAD-7) scales, respectively.

Once all findings have been collected, the Perioperative Management (POM) board will conduct an interdisciplinary discussion of the patient’s case, and the individual treatment strategy will be determined by consensus. The POM board comprises essential representatives from anaesthesiology, cardiology, the specialist surgery participating in the patient’s treatment and, in the case of pathological findings in the PHQ-9 score (> 14), a psychosomatic physician. Other invited participants are the patient’s general practitioner and the practicing specialist, whose attendance is generally optional. The POM board will be held as a blended meeting, with participants able to attend in-person or online, with the latter especially relevant for ambulatory care physicians, who will most likely be unable to attend in person. To fulfil data protection regulations, the POM board meeting will use a special telemedicine software (AMP.clinic; Awesome Technologies, Würzburg, Germany) with a personalised login, where diagnostic findings, such as echocardiography images and sequences, can be shown to all participants.

The following interdisciplinary decisions for individual perioperative patient management are possible: First, cancel surgery if––despite the best possible preoperative optimisation––it would provide no or only little improvement in the patient’s primary disease and quality of life with a high risk of new physical disabilities and comorbidities; such patients will be transferred to conservative treatment. Second, postpone surgery to further optimise the patient preoperatively, which can be recommended individually according to the findings of the preoperative evaluation. This decision might include, for example, establishing guideline-recommended medical HF therapy and up-titration or cardiac rhythm management, as well as further invasive diagnostic and therapeutic coronary or valve interventions, if necessary. Third, proceed with surgery as scheduled in all other cases, and the POM board may recommend additional individual perioperative strategies, such as intraoperative echocardiography or measurement of the pulmonary artery and capillary wedge pressure.

If no individual recommendations are given, the following strategies will be used for all patients in the intervention group. Arterial blood pressure and dynamic preload parameters, such as pulse pressure or stroke volume variation, will be assessed continuously, either non-invasively or invasively, before anaesthesia induction and together with cardiac stroke volume throughout the surgical procedure. Individualised haemodynamic therapy will be derived from these monitoring items, and critical low cardiac output and intraoperative hypotension must be avoided, defined as mean arterial pressure below 65 mmHg, if not individually specified otherwise. Central venous pressure will also be assessed continuously if a central venous catheter is placed, and processed EEG monitoring will be used to avoid burst suppression states. All further intraoperative strategies are at the discretion of the anaesthesiologist and dictated by the surgical requirements.

Postoperatively, structured monitoring for HF-related complications will be performed during the entire hospital stay. Therefore, an interdisciplinary visit at the patient’s bedside will be initiated during the first 3 postoperative days, comprising an anaesthesiologist, cardiologist, the patient’s surgeon and an HF nurse. The HF nurse will perform structured screening for HF complications at the patient’s bedside and the surgical wards daily on weekdays to recognise emerging HF-related complications before they can impede the patient’s recovery process, such as emergency readmission to an intensive care unit because of congestive decompensation. The HF nurse will focus on the patient’s fluid balance to maintain euvolaemia by considering clinical examination, body weight, diuresis, laboratory parameters and vital signs. In cases of uncertainty, the cardiologist supports the nurse. Lastly, the HF nurse will ensure that the patient’s further HF treatment in outpatient care is continued after discharge from the hospital. Therefore, the HF nurse will contact the patient’s general practitioner to provide a summary of the HF-specific findings, further treatment recommendations by the cardiologist, and to arrange a short-term appointment for the patient with the general practitioner.

### Follow-up and end points

Patients will be followed up using a structured telephone interview on postoperative day 30 and a visit to the trial centre on postoperative day 90. The primary end point for postoperative morbidity will be a composite end point, comprising any rehospitalisation, acute kidney injury (AKI), suspected or proven bacterial infection requiring treatment or acute decompensated HF (ADHF) at postoperative day 90. AKI will be defined according to the Kidney Disease: Improving Global Outcomes criteria [12]. ADHF during the index hospital stay is defined as the new onset or worsening of shortness of breath and signs of congestion, including peripheral oedema, moist rales and radiological signs of congestion or pleural effusion requiring intravenous treatment with diuretics. After discharge, ADHF is defined as the new onset or worsening of shortness of breath requiring intravenous diuretic therapy or rehospitalisation for HF > 24 h.

The secondary end points will include the individual components of the primary end point, mortality, the incidence of myocardial infarction or injury, quality of life and patient satisfaction at postoperative days 30 and 90. A detailed list of the end point definitions is provided in the Supplement. Data transferred to patients’ health insurance providers will be analysed to assess their utilisation of healthcare services after discharge and healthcare-related costs in both treatment arms. Physicians involved throughout the interventions, including anaesthesiologists, cardiologists, surgeons and non-clinical physicians, such as patients’ general practitioners, will receive questionnaires to perform a process evaluation of the intervention, which might help identify the strengths and weaknesses of the process, with the latter to be improved before transfer into routine care.

### Sample size calculation and statistical analyses

The primary end point of this trial has not been used in other studies, and no event rates after 90 days were available for our study cohort. However, overall complication rates between 24.6% and 29.7% have been reported for comparable surgical patient cohorts [13–15]. Therefore, our sample size calculation assumed a 26% event rate. Considering a significance level of 0.05, a two-sided *z*-test (PASS 2020; NCSS, LLC, Kaysville, UT, USA) indicated a sample size of 492 per group to demonstrate an odds ratio of 0.65 in the intervention group with a power of 80%. Considering a dropout rate of 10%, 1094 patients with NT-proBNP levels ≥ 450 pg/mL must be included. Since 37% of the patients evaluated in the pilot study had preoperative NT-proBNP levels ≥ 450 pg/mL, and fewer exclusion criteria were applied, we assumed that 30% of all the included patients would have NT-proBNP levels above the cut-off [11]. Therefore, approximately 3647 patients need to be screened to ensure 547 patients in both the control and intervention groups.

An independent clinical end point adjudication committee will evaluate the primary end point of this trial. All primary and secondary end points will be described using descriptive statistics and compared with t-tests or chi-squared tests, depending on the parameter type. Subsequent outcome analyses will use univariable logistic regression for the primary end point, and multivariable logistic regression will be added to adjust for possible confounding variables. Further analyses will evaluate site-specific effects, and if found, adjustments will be performed.

## Discussion

The PeriOP-CARE HF trial will evaluate whether combining systematic preoperative screening using an NT-proBNP-triggered standardised preoperative evaluation with interdisciplinary, multimodal and individually optimised treatment throughout the entire perioperative period will reduce postoperative morbidity in patients aged > 65 years undergoing major surgery with intermediate to high surgical risk according to the current ESC guidelines [3].

We will include a broad spectrum of patients, foremost to be able to detect all potential patients with (unknown) HF since prior studies have shown that a large number of patients undergoing major non-cardiac surgery might suffer from undiagnosed HF, which is detected postoperatively for the first time––when HF-related complications occur [1, 11]. We chose a distinct NT-proBNP cut-off that will be applied to all age groups beyond 65 years. Unfortunately, no specific cut-offs exist for the perioperative period that could have been used. However, the selected cut-off of 450 pg/mL showed good precision in our pilot study, which was conducted before designing the PeriOP-CARE HF trial to select our primary morbidity end point after 30 days. It must be highlighted that the NT-proBNP measurement at the patient’s bedside is a vital part of the new treatment approach that will be evaluated in the PeriOP-CARE HF trial. Since the result will be immediately available, randomisation and preoperative evaluation can be initiated immediately to ensure quick processes leading to a rapid interdisciplinary patient risk discussion by the POM board, and subsequent preoperative optimisation can begin as soon as possible.

To ensure this process, the standardised preoperative evaluation will occur in the anaesthesia outpatient clinic, where all examinations, such as clinical examination, ECG and echocardiography, will be available. It is vitally important to link the elevated NT-proBNP level with potential abnormal ECG and/or echocardiography findings. A relevant number of patients with HF likely suffer from HF with preserved ejection fraction (HFpEF) or atrial fibrillation. Patients with HFpEF are at special risk for postoperative ADHF since 72% of the patients with de novo postoperative ADHF had preserved left ventricular ejection fraction [1]. Patients with atrial fibrillation but not HF might present with elevated NT-proBNP levels comparable to those of patients with HF [16]. However, since perioperative atrial fibrillation is also associated with adverse outcomes after major surgery, the multimodal intervention that will be performed in our trial will be adequate and might improve the outcome for these patients [17].

The POM board has a crucial role in the preoperative optimisation process because all involved specialities will discuss the patients’ individual findings. This discussion will be enriched by the additional expertise and personal knowledge of the patient’s general practitioner. While such a board has never been established in non-cardiac surgery, interdisciplinary conferences, such as tumour boards or the heart teams––which now have a Class I recommendation––have been shown to improve outcomes in other settings through their interdisciplinary decisions [18, 19]. The POM board ensures an individualised decision that is the most suitable and appropriate for the patient and their concomitant diseases. For example, major visceral oncological surgery could not be postponed in a manner an elective knee replacement potentially can. The involved physicians will make all these difficult decisions via consensus to ensure the best possible outcome for the patient.

The intraoperative management of the patients will focus on maintaining optimal haemodynamics, which should help improve patients’ outcomes. Since intraoperative hypotension is strongly associated with adverse outcomes, such as mortality, AKI and myocardial injury, our strategy focuses on avoiding hypotensive episodes [20]. Therefore, we intend to measure arterial pressure continuously from the beginning of anaesthesia induction because many intraoperative hypotension episodes occur during anaesthesia [21]. Consequently, continuous invasive or non-invasive measurement of arterial blood pressure can potentially reduce the incidence and severity of intraoperative hypotension and will, therefore, be performed for all interventional patients participating in the PeriOP-CARE HF trial [22]. Furthermore, patients with HF might be at special risk for volume overload with subsequent congestion in the postoperative period. Therefore, goal-directed therapy will be performed during surgery to ensure optimal oxygen delivery that is guided by fluid boluses, inotropic agents or vasopressors, according to the cardiac stroke volume and dynamic preload parameters.

Goal-directed therapy during non-cardiac surgery has generally been shown to reduce postoperative complications [23, 24]. For example, beneficial effects have been observed concerning mortality, AKI, infective complications and anastomotic leakage after abdominal surgery [25, 26]. However, controversial data have emerged concerning the use of rigid haemodynamic algorithms during major surgery. Especially the goal of maximising cardiac stroke volume and the liberal use of intraoperative dobutamine was not associated with improved outcomes but even adverse effects [27]. Therefore, our trial will use continuous flow monitoring through cardiac stroke volume in all patients and adhere to the current German guidelines on the intraoperative haemodynamic monitoring and management of adults undergoing non-cardiac surgery [28].

The postoperative intervention which is evaluated in the PeriOP-CARE HF trial focuses on the early detection and subsequent treatment of postoperative HF-related complications. This goal is particularly relevant because most postoperative complications occur during the first days after surgery when patients should be under distinct monitoring on the surgical wards. However, HF-related complications might not be the focus of the surgical ward. Therefore, we will perform an interdisciplinary visit to improve the awareness of the surgical ward team for the patients.

In general, HF nurses or nurse practitioners are widely accepted as relevant to improved outcomes in patients with HF [29]. However, they are not currently present on surgical wards, and the PeriOP-CARE HF trial will extend their role to postoperative patients. Furthermore, HF nurses will initiate the patients’ further ambulatory treatment for HF-related issues after discharge, which will be conveyed to their general practitioner before discharge, reducing the period in which a patient’s treatment might be insufficient because the ambulatory physician is unaware of a potential new diagnosis.

The goal of the PeriOP-CARE HF trial is foremost to reducing the postoperative morbidity of the patients receiving the new interdisciplinary treatment approach. Unfortunately, most studies have focused on major adverse cardiovascular events, such as myocardial infarction, stroke and cardiac death. However, we believe these measures do not sufficiently reflect overall risk and, specifically, HF-related complications. Therefore, the primary end point of the PeriOP-CARE HF trial is a composite, including relevant postoperative HF-related morbidity measures. While ADHF is evidently an important end point, any unplanned rehospitalisation during the follow-up period may reflect an additional robust outcome, indicating overall postoperative morbidity. AKI was included in the composite end point because it is generally a frequent postoperative complication that can be reduced by specific interventions. A large proportion of patients develop chronic renal failure after AKI following non-cardiac surgery. Furthermore, the association between HF and chronic or acute kidney failure is well-known [30, 31]. Lastly, any bacterial infection is part of the composite morbidity end point because it represents a specific complication in the postoperative period, and our pilot study revealed a higher incidence in patients with elevated NT-proBNP levels [11]. Interestingly, pneumoniae and other bacterial infections that required antibiotic treatment resulted in an overall increased incidence of any infection. This finding might reflect HF-related limitations in microcirculation and pulmonary congestion, favouring bacterial infection in the lung.

Besides mortality and ischaemic conditions such as myocardial injury and infarction, the secondary end points will focus on the long-term consequences of the interdisciplinary treatment. The proposed intervention will certainly increase the costs of the perioperative treatment, such as the additional point-of-care measurement of NT-proBNP, the POM board and postoperative observation. However, it may provide distinct benefits for both the patient and the healthcare system by reducing the need for procedures (and costs) that might arise from increased long-term postoperative morbidity, such as chronic dialysis. These costs will be examined using health insurance data for the patients included in the PeriOP-CARE HF trial.

Finally, if the intervention evaluated in the PeriOP-CARE HF trial is demonstrated to be beneficial, it could be easily and broadly implemented in the perioperative management of these at-risk patients because most of the hospitals performing surgeries with intermediate to high surgical risk would have already incorporated all the involved specialities. Therefore, implementing the interdisciplinary conference after standardised evaluation is possible. In those hospitals where, for example, no cardiologists are present, telemedicine could be used to bring this expertise to the conference.

In conclusion, the PeriOP-CARE HF trial will evaluate a new interdisciplinary, standardised treatment approach for patients with suspected or known HF to reduce their overall postoperative morbidity. This new treatment approach could potentially reduce not only patients’ morbidity but also healthcare costs. If the PeriOP-CARE HF trial yields positive results, the treatment of patients with HF undergoing major non-cardiac surgery could be considerably improved.

## Supplementary Information

Below is the link to the electronic supplementary material.

Supplementary file1 (DOCX 25 kb)

## Abbreviations

AKI: Acute kidney injury
ADHF: Acute decompensated heart failure
ESC: European Society of Cardiology
ECG: Electrocardiography
GAD-7: Generalized Anxiety Disorder Scale-7
HF: Heart failure
HFpEF: Heart failure with preserved ejection fraction
NT-proBNP: N-terminal pro-brain natriuretic peptide
PHQ-9: Personal Health Questionnaire Depression Scale-9
POM: Perioperative management
