# Supplementary material for: Perioperative interdisciplinary optimisation of patients with heart failure undergoing non-cardiac surgery with intermediate or high surgical risk: the rationale and study protocol for the multicentre, randomised interventional PeriOP-CARE HF trial
Source: Clin Res Cardiol. 2025 Apr 29;114(5):523–31. doi: 10.1007/s00392-025-02626-3 (PMC12058920; doi:10.1007/s00392-025-02626-3)
Supplement: Supplementary file 1 — Supplementary file1 (DOCX 25 kb) [file 392_2025_2626_MOESM1_ESM.docx]

**Supplement**

1. **Primary endpoint**

Composite Morbidity Endpoint (CME) at postoperative day 90

- Any rehospitalisation
- Acute kidney injury
- Acute decompensated heart failure
- Any infection

*See the definitions of the individual CME components below.*

1. **Secondary endpoints**

Secondary endpoints will – if not otherwise specified - be evaluated at postoperative day 30 and 90.

Any rehospitalisation

- Any rehospitalisation for ≥24 hours.

Acute kidney injury

- Definition according to the Kidney Disease: Improving Global Outcomes criteria [1].

Acute decompensated heart failure

- *During the index hospital stay*: New onset or worsening of shortness of breath and signs of congestion, including peripheral oedema, moist rales, and radiological signs of congestion or pleural effusion requiring intravenous treatment with diuretics.
- *After discharge:* New onset or worsening of shortness of breath requiring intravenous diuretic therapy, or rehospitalization for HF >24 hours

Any infection:

- Any suspected or proven bacterial infection requiring specific treatment.

Mortality

- Any death during the observation period.

Myocardial infarction

- Definition according to the fourth universal definition of myocardial infarction [2].

Myocardial injury after non-cardiac surgery (MINS) at postoperative day 1 and 2

- Definition according to the ESC guidelines on cardiovascular assessment and management of patients undergoing non-cardiac surgery [3].

Quality of Life

- Patient Health Questionnaire (PHQ-9)
- 5-level EQ-5D version (EQ-5D-5L)
- Generalized Anxiety Disorder Scale-7 (GAD-7)
- Clinical Frailty Scale (CFS)
- Kansas City Cardiomyopathy Questionnaire (KCCQ-12)

Patient satisfaction

- Patients' Experience Questionnaire (PEQ)

Economic evaluation

- Process data, particularly regarding the implementation of the POM conference
- Process data for cross-sector coordination
- Utilization of services and costs during hospital stay (including intensive care unit and hospital length of stay) and after discharge

1. **References**

1. Stevens PE, Levin A. Evaluation and management of chronic kidney disease: Synopsis of the kidney disease: Improving global outcomes 2012 clinical practice guideline. Ann Intern Med. 2013;158:825–30. doi:10.7326/0003-4819-158-11-201306040-00007

2. van der Wulp K, van Wely M, van Heijningen L, van Bakel B, Schoon Y, Verkroost M, et al. Delirium After Transcatheter Aortic Valve Implantation Under General Anesthesia: Incidence, Predictors, and Relation to Long-Term Survival. J Am Geriatr Soc. 2019;67:2325–30. doi:10.1111/jgs.16087

3. Halvorsen S, Mehilli J, Cassese S, Hall TS, Abdelhamid M, Barbato E, et al. 2022 ESC Guidelines on cardiovascular assessment and management of patients undergoing non-cardiac surgery. Eur Heart J. 2022; doi:10.1093/eurheartj/ehac270
